# Temporal trends, patterns, and predictors of preterm birth in California from 2007 to 2016, based on the obstetric estimate of gestational age

**DOI:** 10.1186/s40748-018-0094-0

**Published:** 2018-12-12

**Authors:** Anura W. G. Ratnasiri, Steven S. Parry, Vivi N. Arief, Ian H. DeLacy, Satyan Lakshminrusimha, Laura A. Halliday, Ralph J. DiLibero, Kaye E. Basford

**Affiliations:** 1 California Department of Health Care Services, Benefits Division, 1501 Capitol Ave, Suite 71.4104, MS 4600, P.O. Box 997417, Sacramento, CA 95899-7417 USA; 2 School of Agriculture and Food Sciences, Faculty of Science, The University of Queensland, Brisbane, QLD 4072 Australia; 3 Department of Pediatrics, University of California Davis, 2516 Stockton Blvd, Sacramento, CA USA; 4 California Department of Health Care Services, Clinical Assurance, and Administrative Support Division, 1501 Capitol Ave, Sacramento, CA 95899-7417 USA; 5 School of Biomedical Sciences, Faculty of Medicine, The University of Queensland, Brisbane, QLD 4072 Australia

**Keywords:** Appropriate for gestational age, Gestational age, Large for gestational age, Low birth weight, Maternal age, Prepregnancy obesity, Preterm birth, Small for gestational age, Maternal smoking, WIC

## Abstract

**Background:**

Preterm birth (PTB) is associated with increased infant mortality, and neurodevelopmental abnormalities among survivors. The aim of this study is to investigate temporal trends, patterns, and predictors of PTB in California from 2007 to 2016, based on the obstetric estimate of gestational age (OA).

**Methods:**

A retrospective cohort study evaluated 435,280 PTBs from the 5,137,376 resident live births (8.5%) documented in the California Birth Statistical Master Files (BSMF) from 2007 to 2016. The outcome variable was PTB; the explanatory variables were birth year, maternal characteristics and health behaviors. Descriptive statistics and logistic regression analysis were used to identify subgroups with significant risk factors associated with PTB. Small for gestational age (SGA), appropriate for gestational age (AGA) and large for gestational age (LGA) infants were identified employing gestational age based on obstetric estimates and further classified by term and preterm births, resulting in six categories of intrauterine growth.

**Results:**

The prevalence of PTB in California decreased from 9.0% in 2007 to 8.2% in 2014, but increased during the last 2 years, 8.4% in 2015 and 8.5% in 2016. Maternal age, education level, race and ethnicity, smoking during pregnancy, and parity were significant risk factors associated with PTB. The adjusted odds ratio (AOR) showed that women in the oldest age group (40–54 years) were almost twice as likely to experience PTB as women in the 20- to 24-year reference age group. The prevalence of PTB was 64% higher in African American women than in Caucasian women. Hispanic women showed less disparity in the prevalence of PTB based on education and socioeconomic level. The analysis of interactions between maternal characteristics and perinatal health behaviors showed that Asian women have the highest prevalence of PTB in the youngest age group (< 20 years; AOR, 1.40; 95% confidence interval (CI), 1.28–1.54). Pacific Islander, American Indian, and African American women ≥40 years of age had a greater than two-fold increase in the prevalence of PTB compared with women in the 20–24 year age group. Compared to women in the Northern and Sierra regions, women in the San Joaquin Valley were 18%, and women in the Inland Empire and San Diego regions 13% more likely to have a PTB. Women who smoked during both the first and second trimesters were 57% more likely to have a PTB than women who did not smoke. Compared to women of normal prepregnancy weight, underweight women and women in obese class III were 23 and 33% more likely to experience PTB respectively.

**Conclusions:**

Implementation of public health initiatives focusing on reducing the prevalence of PTB should focus on women of advanced maternal age and address race, ethnic, and geographic disparities. The significance of modifiable maternal perinatal health behaviors that contribute to PTB, e.g. smoking during pregnancy and prepregnancy obesity, need to be emphasized during prenatal care.

**Electronic supplementary material:**

The online version of this article (10.1186/s40748-018-0094-0) contains supplementary material, which is available to authorized users.

## Background

Preterm birth (PTB), defined as delivery at less than 37 weeks of gestation, occurs in 5 to 18% of pregnancies [1]. Prematurity resulting from PTB may be a result of impaired or slowed fetal intrauterine growth. Worldwide, PTB is a leading cause of infant mortality and the second most common cause of mortality in infants and children under 5 years of age [2]. Preterm infants have a higher mortality rate than infants born at term [2, 3]. Even though the majority of preterm infants now survive, they are at an increased risk for short-term morbidity and long-term neurodevelopmental and health abnormalities including growth impairment [1, 4]. The most common consequences of PTB are learning difficulties, impaired cognition, developmental delay, cerebral palsy, impaired hearing, and impaired vision [4]. In 2005, the estimated annual economic burden associated with PTB in the United States was at least $26.2 billion; this figure continues to increase [3].

The relationship between birth weight and gestational age is a reflection of intrauterine growth. Infants weighing below the 10th percentile of birth weight by sex for a specific completed gestational age of a given reference population are described as small for gestational age (SGA). SGA infants have increased morbidity and an increased risk of mortality in the neonatal period and beyond [3]. The SGA infant is more likely to have neonatal infections, impaired respiratory function, polycythemia, hypoglycemia, jaundice, hypothermia, and impaired ability to feed [3]. These increased complications in SGA infants result in an increased risk of mortality especially among infants who are born both preterm (< 37 completed weeks) [4]. Infants born SGA have also been shown to be at an increased risk of delayed neurodevelopment and poor growth [5]. SGA infants who are born at term (≥37 and < 42 weeks) and preterm have been shown to be 2.4 times and 4.5 times more likely, respectively, to have reduced (stunted) growth in childhood when compared with infants who are born at term with appropriate size for gestational age (AGA) [6].

The National Center for Health Statistics (NCHS) collects vital statistics about gestational age, primarily calculated using the date of the last menstrual period (LMP) [7]. However, the quality of LMP-based dating is controversial, particularly for use in studies where a precise gestational age is an important factor. Imperfect maternal recall, misinterpretation of bleeding early in pregnancy, irregular menstrual cycling, and errors in data entry have been shown to misclassify gestational age, particularly in the evaluation of preterm (< 37 completed weeks) and post-term (≥ 42 weeks) births [7–9].

Some previously published studies have reported that preterm and post-term birth rates are significantly higher in the United States compared to Canada and other industrialized countries, although the relative mortality for preterm compared to term gestation is considerably lower in the United States [10–12]. One speculation is that the rate of PTB is overestimated in the United States, where historically only the LMP was used [12, 13]. European countries calculate gestational age based on the best obstetric estimate (OE), which incorporates an estimate made from the ultrasound findings [12]. Since 2007, a similar OE-based estimate has been included on birth certificates in the state of California.

Because of the increasing evidence for the greater validity of OE-based dating [12], the NCHS began using OE as its standard for the primary measure of gestational age in 2007 [7]. In 2014, the prevalence of PTB in the United States, calculated using OE-based data, was 9.57%, representing an 8% decrease from 2007. The rate of singleton PTB has declined by 10% since 2007 [14].

In 2007, the US Institute of Medicine Committee on Understanding Premature Birth and Assuring Healthy Outcomes recommended that research on PTB should be a public health priority, as more accurate data will provide a better definition of the medical and social problems and risk factors associated with PTB [15]. The Institute noted the need for improved collection of surveillance and descriptive data to more accurately define the nature and scope of the problem [15].

Approximately 4 million women give birth each year in the United States, and 1 in 8 of these births is in California [10]. Therefore, data from the California Birth Statistical Master Files (BSMF) was used to investigate trends in the prevalence of PTB over the 10-year period from 2007, when OE-based data were first included, to 2016. Our aim was to study temporal trends, patterns, and predictors of preterm birth in California from 2007 to 2016, based on the obstetric estimate of gestational age.

In 2014, almost 1 in 10 infants were born preterm in the United States [14]. We therefore defined a secondary aim of this study: to identify significant maternal characteristics and perinatal health behaviors associated with PTB that may be amenable to focused intervention strategies aimed at minimizing PTB. To understand the high-risk subgroups, we extended the analysis to investigate the interactions between maternal age, race and ethnicity, and maternal education. Smoking status during pregnancy, prepregnancy obesity, and maternal demographics were also included, as they have not previously been studied together as risk factors for PTB.

## Methods

### Data source

Data from the California BSMF, compiled by the Center for Health Statistics and Informatics, California Department of Public Health (CDPH), was used for the period 2007 to 2016. The database provided the prevalence of PTB (delivery at < 37 weeks’ gestation), maternal characteristics, and health behaviors across this period.

To identify all PTBs, all live births delivered in the range of 17 to 47 completed weeks of gestation [7], based on the best OE data, with a birth weight of 500 g or greater were included [16]. Births with missing information on gestational age or birth weight were excluded. This retrospective cohort study included 435,280 PTBs as outlined in Additional file 1.

This study was approved by the California Committee for the Protection of Human Subjects (Protocol ID: 16-10-2759) and the California Department of Public Health Vital Statistics Advisory Committee (Project Number: 16-10-2759). Patient consent was waived by the Human Subjects Committee.

### Outcome measure: PTB

The OE-based gestational age was used to define PTB as the outcome variable for this study, by identifying those infants born at less than 37 weeks’ gestation. This was coded as a dichotomous variable (PTB or not). All records for PTB occurring between 17 weeks and 47 weeks of gestation were evaluated. The hospital staff at each delivering institution were responsible for collecting and entering the OE-based gestational age based on the prenatal patient records.

### Explanatory variables

The study analysis included explanatory variables: birth year, maternal sociodemographic characteristics, perinatal health behaviors, health insurance data, participation in perinatal health care, and birth characteristics. Maternal sociodemographic characteristics were added to define the prepregnancy social condition of the women [17], based on self-reported maternal age, education level, race and ethnicity, nativity, and demographic region. To define risk factors that would benefit from intervention, we evaluated perinatal health behaviors including maternal smoking during the first or second trimester, and prepregnancy body mass index (BMI), defined as weight divided by height-squared (kg/m^2^). Using the criteria of the World Health Organization, underweight is classified as a BMI < 18.5 kg/m^2^, normal weight as 18.5 to 24.9 kg/m^2^, overweight as 25.0 to 29.9 kg/m^2^; obesity class I as 30.0 to 34.9 kg/m^2^, obese obesity class II as 35.0 to 39.9, kg/m^2^ and obesity class III as ≥40 kg/m^2^ [18].

The BSMF included self-reported smoking status 3 months before pregnancy and during the first, second, and third trimesters of pregnancy. However, only smoking data from the combination of the first and second trimesters were analyzed.

The BSMF files recorded the type of insurance used: Medi-Cal (public), private insurance, or other types of health insurance. We also added maternal information about whether benefits from the federal Supplemental Nutrition Program for Women, Infants, and Children (WIC) were utilized (household incomes below 185% of the federal poverty line are eligible for WIC program), as receiving such benefits was interpreted as another marker for low income status.

Only Medi-Cal and private-insurance data was examined, because Medi-Cal enrollment is a good indicator of socioeconomic status [19]. We also examined whether women received prenatal care during their first trimester, and included birth characteristics such as parity and plurality in the data analysis.

### Relationship between birth weight and gestational age on intrauterine growth

We grouped birth weight and gestational age into three groups: small for gestational age (SGA) (< 10th percentile), appropriate for gestational age (AGA) (10th to 90th percentile), and large for gestational age (LGA) (> 90th percentile), using new gender specific intrauterine growth curves based on United States data by Olsen et al. (2010) [20]. We also characterized gestational age of 23–41 weeks for singleton births as preterm (< 37 weeks of gestation) and term (≥ 37 weeks of gestation) births. The combination of these two characterizations resulted in six categories of intrauterine growth stage: preterm SGA, preterm AGA, preterm LGA, term SGA, term AGA, and term LGA [3]. Each category was further partitioned into with and without LBW (< 2500 g) infants, as in Ratnasiri et al. (2018) [21].

### Study design and statistical analysis

This retrospective cohort study was performed using data on California resident births from 2007 to 2016. We used descriptive statistics to characterize the prevalence of PTB as the percentage of PTB births in each year, according to maternal characteristics and prenatal health behavior variables.

To identify the maternal characteristics and prenatal health behaviors significantly associated with PTB, we created a series of univariate logistic regression models. First, univariate logistic regression models for the outcome measure of PTB against each explanatory variable were created. Based on model significance testing using the likelihood ratio, and based on consideration of the clinical implications, explanatory variables in multiple logistic regression models were included. We used the stepwise backward elimination method to eliminate variables with a *p* value > 0.05.

This study investigated predictors of preterm birth using multiple logistic regression on singleton births. In the case of cohort studies, some women have delivered more than once during the study period. For these women, there is obviously a time gap between each pregnancy. Maternal age is a well-known predictor of birth outcomes, and socio-behavioral and health conditions of women change over time. Therefore, each pregnancy was individually examined, and PTB outcomes modelled accordingly. The multivariate logistic models were adjusted with birth year and parity as explanatory variables.

The analysis was extended to study the prevalence of PTB according to two different interaction scenarios: the interaction between maternal age and maternal race and ethnicity, and the interaction between maternal education level and maternal race and ethnicity. Multivariate logistic regression modeling was used to study these interactions after controlling for appropriate confounding variables. Multivariate modeling was stratified by maternal age and maternal education level to elaborate disparities in race and ethnicity and to identify the high-risk subgroups. The calculated adjusted odds ratios (AORs) with 95% confidence intervals (95% CIs) and *p* values are presented in the attached tables.

The logistic regression models were restricted to singleton births. All analyses were conducted using SAS software, version 9.3 (SAS Institute Inc., Cary, NC, USA).

## Results

From 2007 to 2016, there were 5,137,376 resident live births documented in the California Birth Statistical Master Files. A total of 30,287 births (0.6%) were excluded due to missing or invalid data for gestational age and birth weight, leaving 5,107,089 births for evaluation. In this population, a total of 435,280 PTBs (Additional file 1) occurred from 2007 to 2016. The number of PTBs, calculated from the OE, decreased from 50,108 in 2007 to 41,555 in 2016 (Table 1). The prevalence of PTB in California decreased from 9.0% (2007) to 8.2% (2014) then increased in the last 2 years to 8.4% (2015) and 8.5% (2016). The multivariate adjusted decline over the study period was 7.0% (AOR, 0.93; 95% CI, 0.91–0.94) (Table 2).

**Table 1:** Total number of births and preterm births and prevalence of preterm birth (as a percentage) according to maternal characteristics and perinatal health behaviors in California from 2007 to 2016

Year	2007	2008	2009	2010	2011	2012	2013	2014	2015	2016
Number of births	554,689	546,201	524,058	508,124	500,367	502,124	492,921	500,404	490,383	487,818
Number of preterm births	50,108	48,796	45,394	42,909	42,080	41,839	40,885	40,767	40,947	41,555
Percentage of preterm births	9.0	8.9	8.7	8.4	8.4	8.3	8.3	8.2	8.4	8.5
Variable										
Maternal age (years)										
< 20	9.1	8.8	8.4	8.2	8.2	8.1	8.0	8.0	8.0	8.3
20–24	8.1	8.0	7.7	7.6	7.4	7.4	7.4	7.1	7.5	7.7
25–29	8.2	8.1	7.8	7.6	7.6	7.6	7.6	7.3	7.5	7.7
30–34	9.0	8.9	8.6	8.4	8.4	8.3	8.1	8.0	8.2	8.2
35–39	10.7	10.6	10.4	10.0	10.0	9.7	9.7	9.5	9.7	9.9
40–54	14.2	14.4	14.1	13.5	13.6	13.3	13.2	13.2	13.5	13.6
Maternal race and ethnicity										
Hispanic	8.6	8.4	8.2	8.0	8.1	8.1	8.2	8.2	8.4	8.7
White^a^	9.0	8.8	8.5	8.2	8.0	7.6	7.7	7.3	7.5	7.6
Asian^b^	8.8	8.8	8.8	8.3	8.4	8.3	8.0	7.5	7.8	8.1
Pacific Islander^c^	10.7	10.3	9.4	10.3	8.6	8.8	8.9	9.1	10.8	9.6
African American	12.7	13.0	12.3	12.2	11.7	11.9	11.6	11.9	11.7	11.4
Multiple race	9.8	9.1	9.2	9.1	8.6	9.1	8.7	9.0	8.9	9.3
American Indian^d^	10.4	10.6	9.3	10.0	8.8	8.8	10.0	8.9	10.1	10.3
Other/unknown^e^	12.8	13.0	11.7	12.2	12.0	11.3	11.1	11.6	10.9	10.5
Maternal education level										
Less than high school diploma	8.8	8.7	8.5	8.3	8.4	8.4	8.7	9.0	9.3	9.5
High school diploma	8.9	9.0	8.5	8.4	8.4	8.2	8.2	8.1	8.3	8.6
Some college or associate degree	9.4	9.1	8.8	8.8	8.7	8.5	8.5	8.3	8.5	8.6
Bachelor’s degree or higher	8.9	8.7	8.5	8.0	7.9	7.9	7.6	7.3	7.5	7.6
Unknown	10.9	11.3	10.6	10.5	10.4	10.2	10.5	10.2	9.8	10.0
Maternal nativity										
Foreign-born	8.2	8.2	8.0	7.8	8.0	7.9	7.9	7.8	8.1	8.4
United States-born	9.7	9.5	9.1	8.9	8.7	8.6	8.5	8.3	8.5	8.6
Maternal demographic region										
Central Coast	8.1	8.1	7.7	7.7	7.7	7.4	7.8	7.4	7.9	7.9
Greater Bay Area	8.5	8.6	8.6	8.4	8.1	8.4	8.2	7.9	8.1	8.3
Inland Empire	9.5	9.0	8.9	8.6	8.4	8.7	8.5	8.3	8.5	8.8
Los Angeles County	9.3	9.3	8.8	8.6	8.7	8.5	8.5	8.4	8.7	8.8
Northern and Sierra	8.4	8.0	7.6	7.4	7.6	7.5	7.7	7.4	8.0	8.1
Orange County	8.7	8.3	8.5	8.1	8.2	7.8	7.6	7.3	7.5	7.9
Sacramento area	8.8	8.5	8.3	8.2	8.1	8.0	7.9	8.1	8.0	8.1
San Diego area	9.5	9.0	9.0	8.4	8.4	8.3	8.1	8.2	8.0	8.4
San Joaquin Valley	9.4	9.7	9.1	8.9	9.0	8.8	8.9	8.8	9.0	9.0
Source of prenatal care payment										
Private	9.2	9.0	8.8	8.7	8.5	8.4	8.2	8.1	8.2	8.3
Medi-Cal	8.7	8.6	8.3	8.1	8.1	8.2	8.3	8.2	8.5	8.7
WIC Participation										
No	9.5	9.4	9.1	8.9	8.7	8.6	8.4	8.1	8.3	8.4
Yes	8.6	8.5	8.3	8.0	8.2	8.1	8.2	8.2	8.4	8.6
First trimester prenatal care initiation										
No	9.1	8.6	8.3	8.3	8.2	8.2	8.2	8.1	8.2	8.5
Yes	8.9	8.9	8.6	8.4	8.4	8.3	8.2	8.1	8.3	8.4
Parity										
Primiparous	8.4	8.2	8.0	7.8	7.7	7.7	7.7	7.4	7.7	7.8
Multiparous (2–5)	9.3	9.2	8.9	8.7	8.6	8.5	8.4	8.4	8.5	8.7
Multiparous (6–12)	14.9	15.2	15.0	14.6	13.9	14.6	14.7	15.3	15.3	15.6
Plurality										
Singleton births	8.0	7.9	7.5	7.4	7.3	7.3	7.2	7.1	7.3	7.5
Multiple births	60.9	59.2	59.0	57.4	57.4	56.0	55.0	54.4	55.8	56.8
Maternal smoking during both first and second trimesters										
No	8.9	8.8	8.6	8.4	8.3	8.2	8.2	8.1	8.2	8.4
Yes	15.7	15.0	14.6	14.2	14.8	15.6	16.4	15.5	16.9	17.9
Prepregnancy body mass index (kg/m^2^)										
Underweight (≤18.5)	9.7	10.0	9.4	9.0	8.9	8.8	8.6	8.5	8.4	8.7
Normal (18.5–24.9)	8.5	8.4	8.1	7.9	7.9	7.7	7.7	7.4	7.5	7.7
Overweight (25.0–29.9)	8.8	8.5	8.3	8.1	8.3	8.2	8.1	8.1	8.2	8.6
Obese I (30.0–34.9)	9.6	9.3	9.1	9.0	8.9	8.8	8.9	9.0	9.3	9.3
Obese II (35.0–39.9)	10.1	10.1	10.4	9.4	9.1	10.1	9.4	9.6	10.1	10.1
Obese III (≥ 40)	10.8	11.2	10.0	11.2	10.3	10.1	10.4	10.0	10.6	10.4

All live births were delivered at 17–47 completed weeks of gestation based on obstetric estimates and birthweight of ≥500 g at birth. Births with missing information on gestational age or birthweight were excluded

Total number of births = 5,107,089

Race/ethnicity results were tabulated using the following race/ethnic groups: Hispanic, White, Asian/ Pacific Islander, African American, Multiple race (two or more races), American Indian, and other

Hispanic origin was determined first and could include any race group. Next, members of the two or more races group were identified and are not reported in the single-race groups. To remain consistent with the population data obtained from the California Department of Finance, the single-race groups are defined as follows:

^a^“White” race group includes White women

^b^“Asian” race group includes Asian Indian, Asian (specified/unspecified), Cambodian, Chinese, Filipino, Hmong, Japanese, Korean, Laotian, Thai, and Vietnamese women

^c^“Pacific Islander” race group includes Guamanian, Hawaiian, Samoan, and other Pacific Islander women

^d^“American Indian” race group includes Aleutian, American Indians, and Eskimo women

^e^“Other/unknown” includes not stated or unknown

**Table 2:** Crude and adjusted odds ratios (with 95% confidence intervals in parentheses) for the prevalence of preterm birth according to birth year and maternal characteristics in California for the period 2007–2016

Variable	Crude odds ratio		Adjusted odds ratio	
	OR (95% CI)	*p*-value^a^	OR (95% CI)	*p*-value^a^
Birth year				
2008	0.99 (0.98–1.00)	0.148	0.99 (0.97–1.00)	0.110
2009	0.95 (0.93–0.96)	<.001	0.94 (0.92–0.96)	<.001
2010	0.93 (0.91–0.94)	<.001	0.92 (0.91–0.94)	<.001
2011	0.92 (0.90–0.93)	<.001	0.91 (0.90–0.93)	<.001
2012	0.92 (0.90–0.93)	<.001	0.91 (0.90–0.93)	<.001
2013	0.91 (0.89–0.92)	<.001	0.90 (0.89–0.92)	<.001
2014	0.89 (0.87–0.90)	<.001	0.89 (0.87–0.90)	<.001
2015	0.92 (0.90–0.93)	<.001	0.91 (0.89–0.93)	<.001
2016	0.94 (0.92–0.95)	<.001	0.93 (0.91–0.94)	<.001
2007	Ref^b^		Ref^b^	
Maternal age (years)				
< 20	1.16 (1.15–1.18)	<.001	1.07 (1.05–1.09)	<.001
25–29	0.97 (0.96–0.98)	<.001	1.08 (1.07–1.10)	<.001
30–34	1.01 (1.00–1.02)	0.015	1.24 (1.22–1.26)	<.001
35–39	1.20 (1.19–1.22)	<.001	1.51 (1.49–1.54)	<.001
40–54	1.54 (1.52–1.57)	<.001	1.92 (1.88–1.96)	<.001
20–24 (ref)	Ref^b^		Ref^b^	
Maternal race/ethnicity				
African American	1.77 (1.74–1.79)	<.001	1.64 (1.61–1.67)	<.001
American Indian	1.46 (1.39–1.54)	<.001	1.26 (1.18–1.35)	<.001
Asian	1.16 (1.14–1.17)	<.001	1.40 (1.38–1.42)	<.001
Hispanic	1.23 (1.22–1.24)	<.001	1.22 (1.21–1.24)	<.001
Multiple Race	1.26 (1.23–1.29)	<.001	1.23 (1.20–1.26)	<.001
Other Unknown	1.35 (1.32–1.38)	<.001	1.26 (1.22–1.30)	<.001
Pacific Islander	1.48 (1.41–1.55)	<.001	1.43 (1.35–1.51)	<.001
White (ref)	Ref^b^		Ref^b^	
Maternal education				
< High school	1.37 (1.36–1.38)	<.001	1.35 (1.33–1.38)	<.001
High school diploma	1.28 (1.27–1.29)	<.001	1.30 (1.28–1.31)	<.001
Some college/associate degree	1.27 (1.26–1.29)	<.001	1.28 (1.26–1.30)	<.001
Unknown	1.39 (1.36–1.41)	<.001	1.30 (1.27–1.34)	<.001
Bachelor’s degree or higher (ref)	Ref^b^		Ref^b^	
Maternal nativity				
United States-born	1.08 (1.07–1.08)	<.001	1.15 (1.14–1.16)	<.0001
Foreign-born (ref)	Ref^b^		Ref^b^	
Maternal demographic region				
Central Coast	0.97 (0.95–1.00)	0.023	1.01 (0.98–1.04)	0.621
Greater Bay Area	1.03 (1.00–1.05)	0.022	1.04 (1.01–1.06)	0.007
Inland Empire	1.14 (1.11–1.16)	<.001	1.13 (1.10–1.16)	<.001
Los Angeles County	1.13 (1.11–1.15)	<.001	1.12 (1.09–1.14)	<.001
Orange County	0.99 (0.97–1.02)	0.493	1.05 (1.02–1.08)	0.001
Sacramento Area	1.03 (1.01–1.06)	0.016	1.05 (1.02–1.08)	0.001
San Diego Area	1.07 (1.04–1.09)	<.001	1.13 (1.10–1.16)	<.001
San Joaquin Valley	1.21 (1.18–1.24)	<.001	1.18 (1.15–1.22)	<.001
Northern and Sierra (ref)	Ref^b^		Ref^b^	
Source of prenatal care payment				
Medi-Cal (Public)	1.13 (1.12–1.14)	<.001	1.12 (1.11–1.13)	<.001
Private insurance (ref)	Ref^b^		Ref^b^	
^c^WIC participation				
No	0.95 (0.94–0.96)	<.001	1.13 (1.12–1.14)	<.001
Yes (ref)	Ref^b^		Ref^b^	
First trimester prenatal care initiation				
No	1.08 (1.07–1.09)	<.001	1.07 (1.06–1.09)	<.001
Yes (ref)	Ref^b^		Ref^b^	
Parity				
Primiparous	1.03 (1.02–1.04)	<.001	1.16 (1.15–1.17)	<.001
Multiparous 6–12	1.84 (1.80–1.88)	<.001	1.35 (1.32–1.39)	<.001
Multiparous 2–5 (ref)	Ref^b^		Ref^b^	
Maternal smoking				
Yes	1.85 (1.81–1.89)	<.001	1.57 (1.53–1.62)	<.001
No (ref)	Ref^b^		Ref^b^	
Maternal prepregnancy body mass index (kg/m^2^)				
Underweight, < 18.5	1.23 (1.20–1.25)	<.001	1.23 (1.20–1.25)	<.001
Overweight, 25.0–29.9	1.09 (1.08–1.09)	<.001	1.06 (1.05–1.07)	<.001
Obese I–30.0–34.9	1.22 (1.21–1.23)	<.001	1.16 (1.15–1.17)	<.001
Obese II, 35.0–39.9	1.31 (1.29–1.33)	<.001	1.24 (1.22–1.26)	<.001
Obese III, ≥ 40	1.44 (1.42–1.47)	<.001	1.33 (1.30–1.36)	<.001
Normal, 18.5–24.9 (ref)	Ref^b^		Ref^b^	

*OR* Odds ratio, *AOR* Adjusted odds ratio, *CI* Confidence interval, *BMI* Body mass index

^a^*p* value determined using the χ^2^ test

^b^*Ref* Reference group

^c^*WIC* Supplemental Nutrition Program for Women, Infants, and Children (household incomes below 185% of the federal poverty line are eligible for WIC)

All singleton live births were delivered at 17–47 completed weeks of gestation based on obstetric estimates and birthweight of ≥500 g at birth. Births with missing information on gestational age or birthweight were excluded

Overall, there were highly significant (*p* < .001) differences in the prevalence of PTB, compared with the reference groups, for each maternal sociodemographic characteristic and perinatal health behavior studied: maternal age, race and ethnicity, education level, nativity, demographic region, smoking status, prepregnancy BMI, source of prenatal health care insurance payment, use of first-trimester prenatal care, and parity (Table 2).

### Maternal age

The mean maternal age in the selected population increased from 28.0 years in 2007 to 29.6 years in 2016 in California (data not presented).

**Fig. 1:**
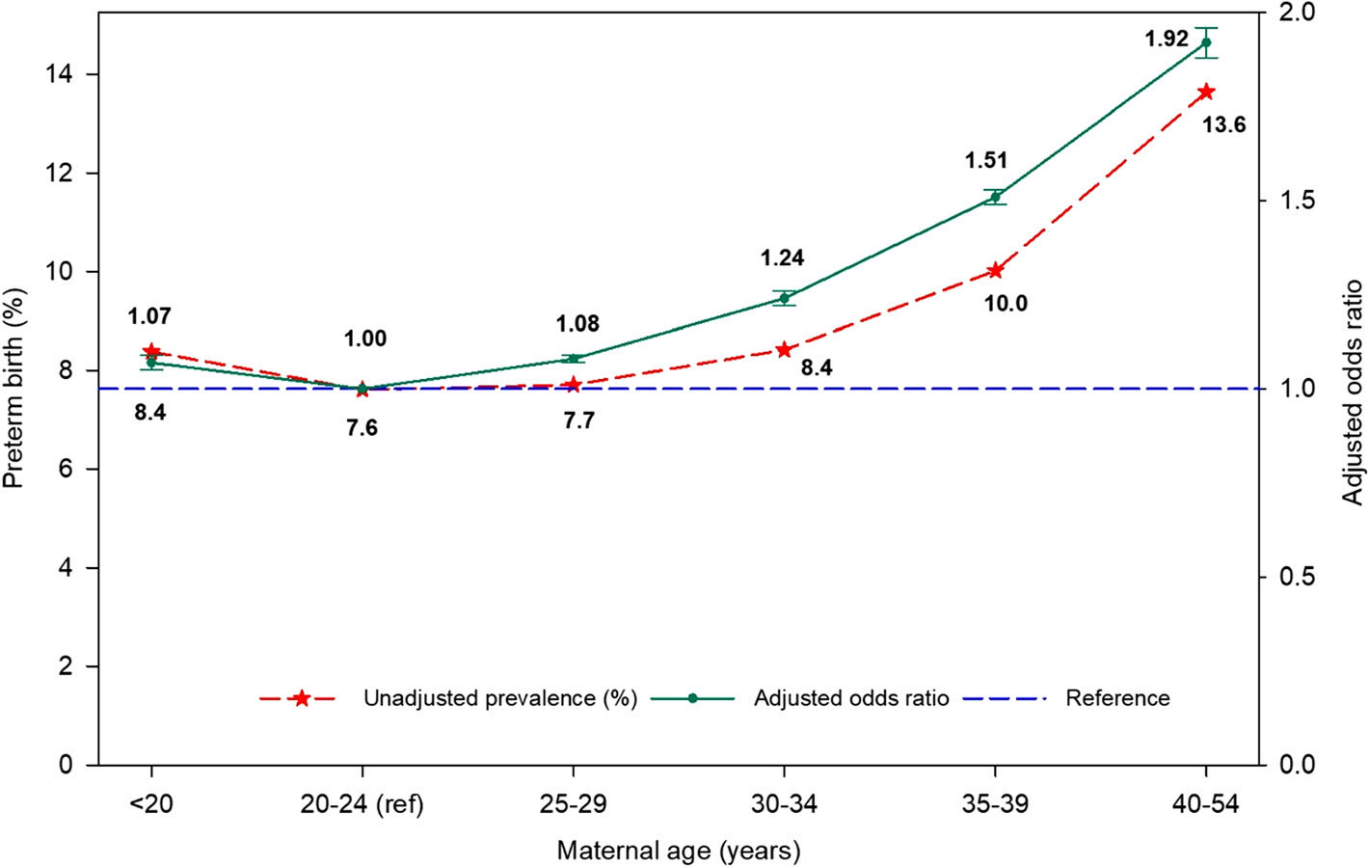
Unadjusted prevalence of and adjusted odds ratio for preterm singleton birth for each maternal age group in California for the period 2007–2016

From 2007 to 2016, the prevalence of PTB (Table 1) declined by over 5% in all age groups, except for the 40- to 54-year age group, which saw a 4.3% decrease. The largest decline was seen in teenagers, who had a 9.1% drop, and the smallest decline was seen in the oldest age group, which had a decrease of 4.3%. There were persistent disparities between the maternal age groups during all years of the study period (Table 1, Fig. 1).

Women in the oldest age group (40–54 years) were 92% more likely to have a PTB compared with women in the 20- to 24-year reference age group (AOR, 1.92; 95% CI, 1.88–1.96) (Table 2). Women in the 35- to 39-year age group were 51% more likely to have a PTB compared with women in the reference age group (AOR, 1.51; 95% CI, 1.49–1.54), while women in the 30- to 34-year age group had a 24% greater chance of a PTB (AOR, 1.24; 95% CI, 1.22–1.26) (Table 2).

### Maternal race and ethnicity

The prevalence of PTB declined by over 5% from 2007 to 2016, except in women of Hispanic (− 0.1%) and American Indian (0.9%) race and ethnicity (Table 1). The largest decline (15%) occurred among white women, from 9.0% in 2007 to 7.6% in 2016. The second largest decline (10.3%) was in African American women, from 12.7% in 2007 to 11.4% in 2016. During the study period, the prevalence of PTB varied substantially between racial and ethnic groups in California. In 2016, the overall rate of PTB was 8.5% (Table 1). However, in that year 11.4% of infants born to African American women were preterm, compared with 7.6% of those born to white women and 7.6% of those born to Asian women (Table 1). From 2007 to 2016, the rate of PTB among African American women declined at a slower pace (10.3%) than in white women (15.0%). Almost 50% of births were in Hispanic women, but unlike other racial and ethnic groups, the prevalence of PTB among Hispanic women increased slightly, from 8.6% in 2007 to 8.7% in 2016.

As shown in Table 2, there were significant disparities in the prevalence of PTB between women in different racial and ethnic groups. African American women had a persistent 64% greater prevalence of PTB throughout the study period compared with white women, who were defined as the reference group (AOR, 1.64; 95% CI, 1.61–1.67). There were 43% more PTBs in Pacific Islanders (AOR, 1.43; 95% CI, 1.35–1.51), 40% more in Asian women (AOR, 1.40; 95% CI, 1.38–1.42), and 22% more in Hispanic women (AOR, 1.22; 95% CI, 1.21–1.24) (Table 2).

### Interaction between maternal age and maternal race and ethnicity

Although the prevalence of PTB differed considerably by maternal age and race-ethnicity (Tables 1 and 2), this information provides little insight into how the risk for PTB may differ between younger and older women within each racial and ethnic group or across racial and ethnic groups. Therefore, to identify whether the relationship between PTB and maternal age was consistent across racial and ethnic groups, we pooled data from 2007 to 2016 and investigated the effect of maternal age and race and ethnicity on the unadjusted prevalence of PTB.

**Fig. 2:**
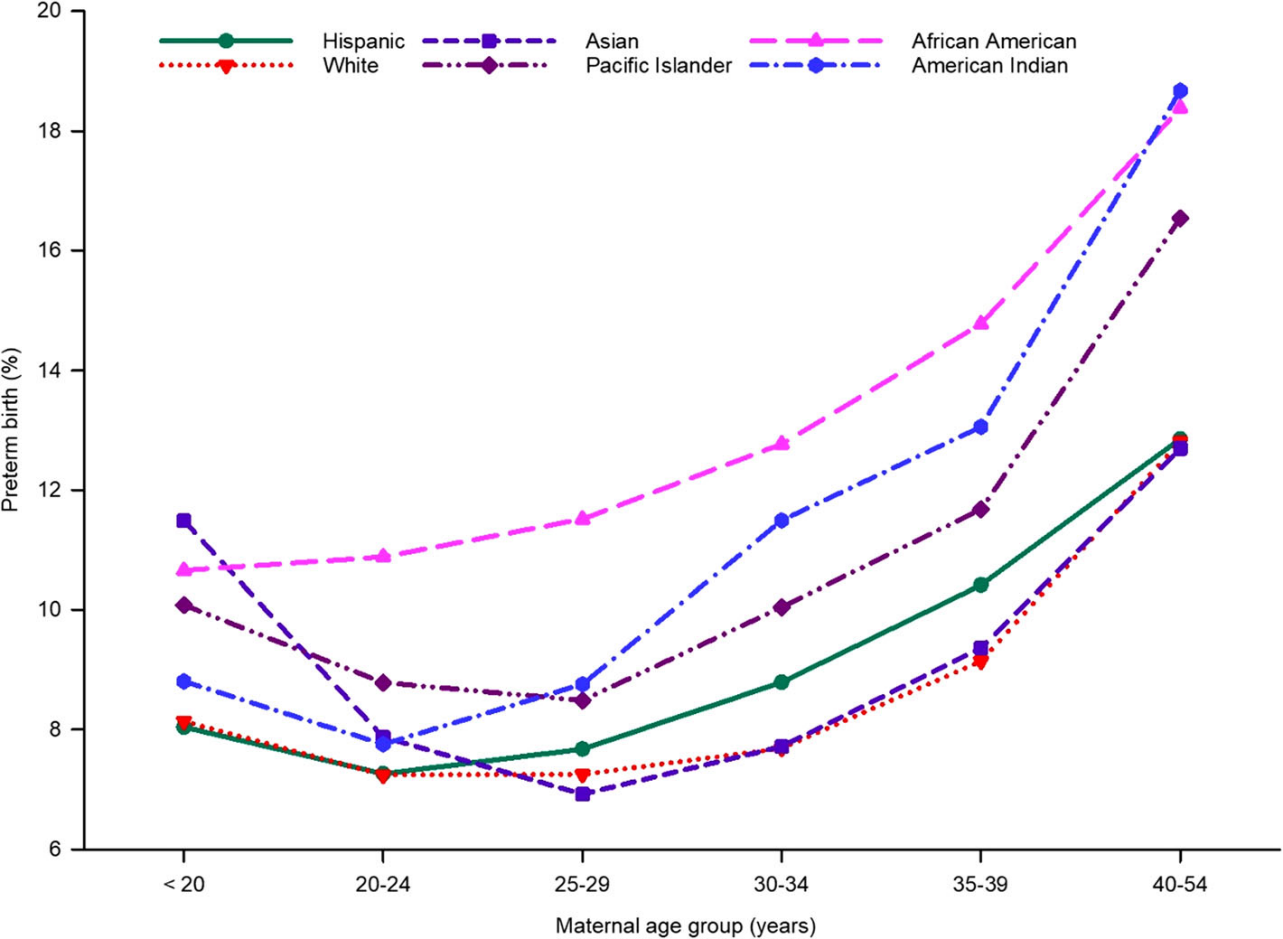
Unadjusted prevalence of preterm birth for maternal age group by maternal race and ethnicity in California for the period 2007–2016

Different patterns were observed across maternal age groups for each race and ethnicity studied (Fig. 2); that is, the response pattern of prevalence of PTB across maternal age groups was not consistent across racial and ethnic groups. The women at the younger and older ends of the childbearing age range were at increased risk for PTB. This finding is consistent with that of a national study that used pooled data for the 1998 to 2000 United States birth cohort from the NCHS [15].

Unlike the other racial and ethnic groups, where a dip in the prevalence of PTB was observed for mothers aged from 20 to 29 years, the prevalence of PTB increased with age for African American women (Fig. 2). The prevalence of PTB in African American women was 10.7% in the age group 20 years and younger and 18.4% in the 40- to 54-year age group. There was a clear gap in the prevalence of PTB between African American and white women at every age group.

Asian women had the highest prevalence of PTB when they gave birth at less than 20 years of age (11.5%). However, the prevalence of PTB decreased markedly in the next two age groups (20–24 years and 25–29 years). For the 25–29 year age group, the rate of PTB for Asian women remained the lowest of all racial and ethnic groups (6.9%), although their rate did increase with age, consistent with other racial and ethnic groups (Fig. 2).

American Indian (18.7%) and African American (18.4%) women in the 40- to 54-year age group were at particular risk for PTB. African American women showed a rapid increase in the prevalence of PTB at 20 years of age or older and had the highest prevalence of PTB (18.4%) when they gave birth at 40 years of age or older (Fig. 2). Births to Pacific Islander women 30 years of age or older showed an increased prevalence of PTB.

Even after adjusting for all possible confounders available in the BSMF dataset, we found that Asian women had the highest prevalence of PTB in the age group less than 20 years (AOR, 1.40; 95% CI, 1.28–1.54) (Fig. 2, Additional file 2).

Consistent with the observed prevalence of PTB, the adjusted ORs showed that Pacific Islander, American Indian, and African American women aged 40 to 54 years were twice as likely to have a PTB compared with women aged 20 to 24 years (Fig. 2, Additional file 2).

### Maternal education level

The prevalence of PTB differed according to maternal education level, although these differences were not as large as those observed for maternal age and racial-ethnic group (Table 1). Compared with the reference group of women with a bachelor’s degree or higher, women with less than a high-school education had a 35% greater chance of having a PTB (AOR, 1.35; 95% CI, 1.33–1.38), followed by women with a high-school diploma at 30% (AOR, 1.30; 95% CI, 1.28–1.31) (Table 2).

### Interaction between maternal education level and maternal race and ethnicity

**Fig. 3:**
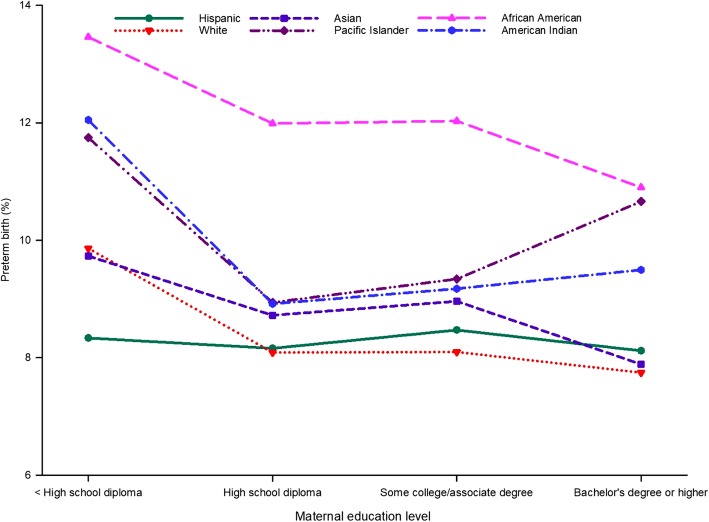
Unadjusted prevalence of preterm birth for maternal education level by maternal race and ethnicity in California for the period 2007–2016

Figure 3 shows the unadjusted prevalence of infants born preterm according to maternal race and ethnicity and education level. The unadjusted prevalence of PTB was relatively high among women in all racial and ethnic groups with less than a high-school diploma, except in Hispanic women (Fig. 3).

Based on the unadjusted prevalence of PTB, infants born to African American women consistently had the highest prevalence of PTB for all levels of education. However, the prevalence of PTB in this population decreased with increasing level of education, as with Asian and white women. The unadjusted prevalence of PTB was consistently low for Hispanic women, while all other races and ethnicities showed larger disparities in the prevalence of PTB across education levels. For all ethnicities, the PTB rate was the highest for women who did not have a high-school diploma.

Pacific Islander and American Indian women showed a relatively higher prevalence of PTB when they had either some college education, or an associate degree or higher. In contrast, white, Hispanic, Asian, and African American women with a bachelor degree or higher showed a lower prevalence of PTB.

Additional file 3 shows the results of multivariate analysis controlling for the confounding effects of maternal education level and race and ethnicity. American Indian women (AOR, 1.69; 95% CI, 1.26–2.26), those of multiple race (AOR, 1.60; 95% CI, 1.42–1.80), and Pacific Islanders (AOR, 1.60; 95% CI, 1.24–2.07) were at risk for PTB with an education level less than a high-school diploma, compared with women who had a bachelor degree or higher (Additional file 3).

### Maternal nativity

From 2007 to 2016, the number of PTBs increased by 2.0% for foreign-born women and declined by 11.5% for US-born women (Table 1). However, US-born women were 15% more likely to have a PTB when compared with foreign-born women (AOR, 1.15; 95% C1, 1.14–1.16) (Table 2).

### Maternal geographic region

**Fig. 4:**
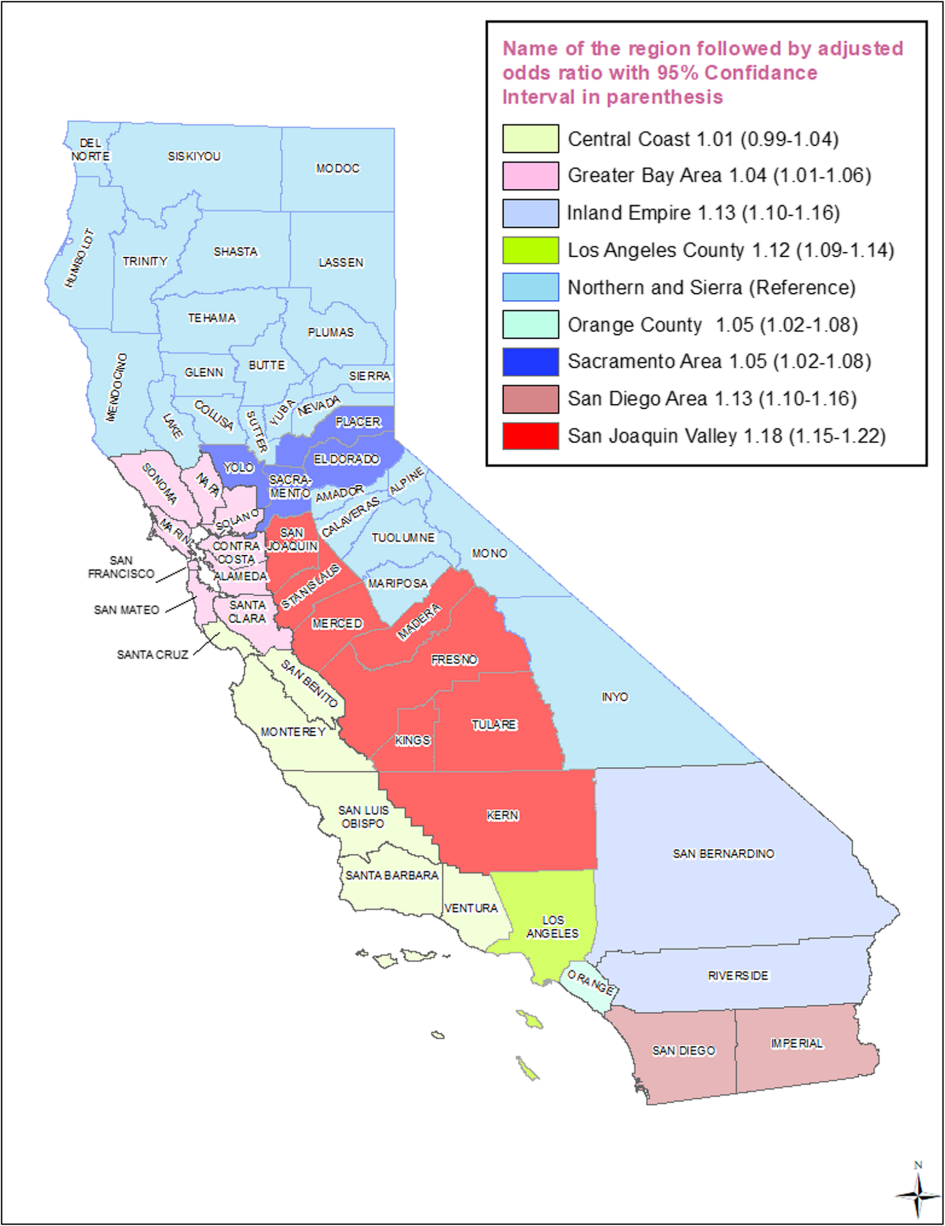
Adjusted odds ratio (with 95% confidence interval in parenthesis) for the prevalence of preterm birth by each demographic region identified on a map of California for the period 2007–2016. Northern and Sierra region is the reference group

Almost 26% of California births occurred in Los Angeles County, followed by the Greater Bay Area (17%) and San Joaquin Valley (13%) (data not presented). From 2007 to 2016, the prevalence of PTB among women residing along the Central Coast (2.9%) and in the San Joaquin Valley (3.9%) declined at a slower rate than in the other regions (Table 1). Women in the San Joaquin Valley were 18% more likely to have a PTB, followed by those in the Inland Empire (13%) and San Diego (13%) regions, compared with those in the Northern and Sierra regions (Table 2, Fig. 4).

### Insurance type and first-trimester initiation of prenatal care

Women dependent on Medi-Cal as their source of prenatal care payment were 12% more likely to have a PTB compared with women with private insurance (AOR, 1.12; 95% CI, 1.11–1.13) (Table 2). Women who did not participate in the Women, Infants, and Children (WIC) program were at risk for PTB (AOR, 1.13; 95% CI, 1.12–1.14). Women who did not participate in prenatal care during the first trimester were 7% more likely to have a PTB compared with women who initiated prenatal care during the first trimester (AOR, 1.07; 95% CI, 1.06–1.09) (Table 2).

### Birth characteristics

Primiparous women were 16% more likely to have a PTB, compared with women who were multiparous with 2 to 5 infants (AOR, 1.16; 95% CI, 1.15–1.17). Women who were multiparous with 6 to 12 infants were 35% more likely to have a PTB than women who were multiparous with 2 to 5 infants (AOR, 1.35; 95% CI, 1.32–1.39) (Table 2).

### Maternal health behavior

Maternal smoking during the first and second trimesters decreased significantly, by 35%, from 2007 to 2016 (data not presented). However, women who smoked during both the first and second trimesters were 57% more likely to have a PTB than women who did not smoke (AOR, 1.57; 95% CI, 1.53–1.62) (Table 2).

Women who were in the obese class III group (BMI ≥ 40 kg/m^2^) were 33% more likely to experience PTB than women of normal weight (BMI 18.5 to 24.9 kg/m^2^) (AOR, 1.33; 95% CI, 1.30–1.36). Underweight (BMI < 18.5 kg/m^2^) women were 23% more likely to experience PTB than women of normal prepregnancy weight (AOR, 1.23; 95% CI, 1.20–1.25) (Table 2). The prevalence of PTB increased with increasing obesity class (Table 2).

### Relationship between birth weight and gestational age on intrauterine growth

Table 3 contains the percentage of singleton births at 23–41 weeks of gestation based on obstetric estimates (OE) for the six categories of intrauterine growth stage for the 4,917,260 such singleton births in California from 2007 to 2016, with the percentage of LBW infants in parenthesis.

**Table 3:** Percentage of singleton births at 23–41 weeks of gestation based on obstetric estimates (OE) for the six categories of intrauterine growth stage for the 4,917,260 such singleton births in California from 2007 to 2016, with the percentage of LBW infants in parenthesis

Category	2007	2008	2009	2010	2011	2012	2013	2014	2015	2016
Preterm-SGA	0.4 (100)	0.4 (100)	0.4 (100)	0.5 (100)	0.4 (100)	0.4 (100)	0.4 (100)	0.4 (100)	0.4 (100)	0.5 (100)
Preterm-AGA	5.8 (47.9)	5.8 (47.4)	5.6 (48.8)	5.5 (49.5)	5.5 (49.9)	5.5 (49.6)	5.4 (50.7)	5.3 (49.9)	5.5 (50.6)	5.6 (49.9)
Preterm-LGA	1.2 (17.0)	1.1 (17.3)	1.0 (17.6)	1.0 (19.5)	0.9 (19.6)	0.9 (20.3)	0.9 (21.7)	0.9 (19.9)	0.9 (20.4)	0.9 (19.7)
Term-SGA	4.9 (29.4)	4.8 (29.0)	5.0 (28.7)	5.0 (29.0)	5.0 (28.2)	4.9 (28.2)	5.0 (28.2)	5.0 (27.7)	5.0 (28.0)	4.9 (27.9)
Term-AGA	81.1 (0.4)	81.2 (0.4)	81.6 (0.4)	81.8 (0.4)	82.0 (0.5)	82.0 (0.5)	82.2 (0.5)	82.3 (0.5)	82.3 (0.5)	82.0 (0.5)
Term-LGA	6.7 (0.0)	6.6 (0.0)	6.4 (0.0)	6.3 (0.0)	6.3 (0.0)	6.3 (0.0)	6.1 (0.0)	6.1 (0.0)	5.9 (0.0)	6.1 (0.0)
Preterm-SGA + Term-SGA	5.3 (35.2)	5.3 (34.7)	5.4 (34.3)	5.5 (34.8)	5.4 (34.0)	5.3 (34.1)	5.4 (33.9)	5.5 (33.4)	5.4 (33.9)	5.4 (33.9)

Preterm: < 37 weeks of gestation; Term: ≥37 weeks of gestation; *SGA* Small for gestational age, *AGA* Appropriate for gestational age, *LGA* Large for gestational age, *LBW* Low birth weight (< 2500 g)

#### Preterm birth

The percentage of preterm SGA (from 0.4 to 0.5%; *p* = 0.239) and preterm AGA (5.8 to 5.6%; *p* = 0.050) did not change significantly, but preterm LGA (1.2 to 0.9%; *p* = 0.002) declined significantly from 2007 to 2016. Among preterm SGAs, all the births were LBW infants. Interestingly, percent LBW infants among preterm AGAs rose slightly but significantly (*p* = 0.003) from 47.9% in 2007 to 49.9% in 2016. Percent LBW infants among preterm LGAs also rose slightly but significantly (*p* = 0.007) from 17.0% in 2007 to 19.7% in 2016 (Table 3).

#### Term birth

The percentage of term SGA (from 4.9 to 4.9%; *p* = 0.130) did not change significantly, while term AGA (81.1 to 82.0%; *p* < 0.001) increased marginally but significantly from 2007 to 2016. The term LGA (6.7 to 6.1%; p < 0.001) also declined slightly but significantly (Table 3).

Overall, SGA infants (both preterm SGA + term SGA) among singleton births at 23 to 41 weeks based on OE of gestational age did not change significantly (*p* = 0.103) during the study period. However among SGA infants, LBW births declined significantly (p = 0.002) from 35.2% in 2007 to 33.9% in 2016.

## Discussion

To examine temporal trends, patterns, and predictors of PTB in California from 2007 to 2016, based on the obstetric estimate of gestational age, we performed a retrospective population-based cohort study to evaluate the large, highly diverse group of selected resident births in California from 2007 to 2016.

To the best of our knowledge, this is the first study to investigate the temporal trends, patterns, and predictors of PTB in California involving perinatal health behaviors (smoking during pregnancy and prepregnancy BMI) using the OE based gestational age to calculate PTB.

The OE-based estimate, which incorporates all perinatal factors including ultrasound data, is expected to have higher validity than dating based on the LMP, as the latter may be inaccurate because of poor maternal recall and individual variation in menstrual-cycle length [22, 23]. Early ultrasound dating is considered to be the most accurate method of determining gestational age [24]. Prenatal ultrasound performed before 20 weeks of gestation is more accurate (95% CI, ± 3–5 days) than any other prenatal or postnatal estimate of pregnancy duration [25–28].

The findings of this study emphasize the advantage of exploring the interactions between maternal factors, including age, race and ethnicity, and education, to identify subgroups at high risk for PTB. Consistent with the results of national studies [14, 29], the overall percentage of PTB in our study shows a downward trend from 2007 to 2016. The national rate of PTB was 9.57% in 2014, 8% lower than in 2007 [14]. Consistent with national trends, the prevalence of PTB in California declined by 5.6%. The proposed reasons for the decrease in the rate of PTB in the United States during the period from 2006 to 2013 include changes in characteristics and risk factors in the obstetric population, the implementation of specific evidence-based guidelines, novel interventions in women with identifiable risk factors, and improved public health policies and regulations [30]. Although the United States has reduced its rate of PTB, the prevalence of PTB in California shown in the present study indicates that the prevalence in the United States remains among the highest in the world, especially compared with other developed countries [31]. As intrinsic population heterogeneity is likely to play a role in this disparity, employing prevention interventions or reducing known risk factors will have a varying effect [30].

These findings indicate that maternal age is a significant predictor of PTB, as the prevalence of PTB increased with maternal age from 20 years of age for each year of the 2007–2016 study period in California. The prevalence of PTB has declined at a slower rate in women aged 35 years or older, the age group that defines advanced maternal age [32]. Consistent with several previously published studies [33–37], our findings show that maternal age is a significant risk factor for PTB, even after adjusting for confounding variables. We also noted significant disparities in PTB among the maternal age groups studied (Table 1). Several previously published studies have identified young maternal age as an important risk factor for PTB [33, 38, 39]. In 1997, Hediger et al. found that young adolescents, less than 16 years of age at the time of their LMP, had twice the risk for PTB compared with women aged between 18 and 29 years [35]. The risk of PTB has been previously reported to decrease with increasing age in adolescent mothers [15]. It is not known whether the increased risk for PTB among young adolescents relates to the effects of young age on reproductive biology or to the increased prevalence of other risk factors (e.g., smoking, underweight) associated with poor socioeconomic status [33].

In the present study, we noted that births to women aged 35 years or older increased by almost 25.6% from 2007 to 2016 (data not presented), and these women were at increased risk for PTB. These findings are consistent with those of previously published studies [29, 40, 41]. The mean maternal age in the population experiencing PTB in California increased from 28.0 years in 2007 to 29.6 years in 2016. We observed that 19.1% of women 35 years of age or older delivered 24.2% of the infants born at preterm (data not presented). Older women have some common risk factors for PTB such as preexisting chronic conditions, extremes of BMI, and low socioeconomic status [15]. The largest population-based study of PTB conducted so far was undertaken in Sweden. In that study, the reported rate of PTB was 54% higher in women aged 40 to 44 years and 63% higher in women aged 45 years or older, compared with women 20 to 29 years of age [42]. Advanced maternal age is a risk factor for female infertility, pregnancy loss, fetal anomalies, stillbirth, and obstetric complications including chronic hypertension, hypertensive disorders of pregnancy, diabetes, and cesarean delivery [32, 42–44]. The risk for PTB has previously been reported to be increased for women aged older than 35 years, regardless of race or ethnicity [40]. In 2006, Behrman and Butler reported that the risk of PTB was greater for women aged older than 35 years when delivering their first child [15].

Significant disparities in PTB between racial and ethnic groups in the United States and in California are well documented [14, 45]. Our findings are consistent with those of a previous study [46]. We found persistent disparities after accounting for all measured potential confounding variables. The persistent gap between PTB rates in African American and white women in California, and throughout the United States, continues to be a serious public health concern. Although the reasons are not clear, these racial and ethnic disparities have persisted for decades [15]. In 2003, Lu and Halfon suggested explanations including racial differences in socioeconomic status, maternal behavior, stress, infection, and genetic factors [46]. On average, African American women have been shown to be more socioeconomically disadvantaged than white women [47], and a poorer socioeconomic condition is associated with an increased risk for PTB [15]. In our study, Asian and Pacific Islander women in California were also at risk for PTB, as defined using the OE.

Our study shows an increased prevalence of PTB for African American women in all maternal age groups, except mothers less than 20 years of age (Fig. 2). The high values for this ethnic group are consistent with data from a national study of births in the United States from 1998 to 2000 [15]. The gap between African American women and white women increases slightly with maternal age (Fig. 2). In 1996, Geronimus attributed the different rates of increase with age to “weathering,” in which the effects of social inequality on health combine with age to create an increasing gap in health status between African American and white women through young and middle adulthood; this gap can affect reproductive outcomes [48]. However, the evidence supporting the weathering hypothesis remains inconclusive because most studies that use cross-sectional data cannot adequately control for potential cohort effects [15]. However, the findings of the present study, showing an interaction between maternal race and ethnicity, might support the weathering hypothesis.

In the present study, American Indian women giving birth at 35 years of age or older were twice as likely to have a PTB compared with women in the 20- to 24-year age group of the same ethnicity (Fig. 2). Pacific Islander, American Indian, and African American women 40 years of age or older were almost twice as likely to have a PTB as were women in the 20- to 24-year age group of the same ethnicity. Further studies on the interactive effects of maternal age and race and ethnicity on PTB are required to understand the roles of social determinants and maternal age.

Educational attainment is among the most widely used indicators of socioeconomic status because it is an easily defined characteristic [49] and because of its influence on future occupational opportunities and earning potential [50]. Population-based studies that include measures of socioeconomic status have consistently found that the poorest women have the worst pregnancy outcomes [51]. In the present study, women with less than a graduate level of education are 25% more likely to have a PTB than those with a bachelor degree or higher (Table 2). The interaction between maternal age and maternal education level shows large gaps in the prevalence of PTB between these two education groups in the 20- to 24-year-old, 25- to 29-year-old, and 30- to 34-year-old groups (Fig. 3). In contrast, the prevalence of PTB is more similar across educational groups in women 40 years of age or more, suggesting that the influence of advanced maternal age on PTB is independent of education level.

Unlike women of other races and ethnicities, Hispanic women showed less disparity in the prevalence of PTB by education level, suggesting that Hispanic populations tend to demonstrate healthier birth outcomes regardless of socioeconomic status [52]. When compared with Hispanic births, women of all other ethnic groups show wider disparities in the prevalence of PTB by education level. The findings of this study, based on the interaction between maternal race and ethnicity and education level (Fig. 3), found that even with higher educational attainment the prevalence of PTB remains high among women of African American origin, Pacific Islanders, and American Indians; this is possibly inconsistent with the weathering hypothesis. Additional research is needed to understand the risk factors that contribute to the high prevalence of PTB, even with higher educational attainment, in certain racial and ethnic groups.

In this study, foreign-born women were less likely to have a PTB than US-born women (Table 2). In 2002, the US Centers for Disease Control and Prevention reported that foreign-born women have better birth outcomes compared with their US-born racial and ethnic counterparts, despite later initiation of prenatal care and lower education levels [53]. Similar findings are reported from other national- and state-level studies [45, 54, 55]. These findings suggest that, compared with their US-born peers, foreign-born African Americans, Asians, Hispanics, and Filipinos have lower rates of infant mortality, low birth weight, and PTB. It is important to improve access to prenatal care and target the identification of risk factors for PTB among women of advanced maternal age, taking appropriate action to prevent PTB. More research is needed to decrease the risk of PTB at advanced maternal age.

Maternal smoking status and prepregnancy height and weight have only been recorded in the BSMF since 2007; we are the first to report trends in smoking during pregnancy and prepregnancy BMI in the population experiencing PTB. The results of this study provide information on BMI among women of childbearing age at the population level (Table 1). The latest Surgeon General’s Report on Smoking and Health states that tobacco use during pregnancy is a preventable cause of disease and death of the mother, fetus, and infant [56]. Our study shows that women who smoke during pregnancy are more than 50% more likely to deliver preterm (Table 2). Dietz et al., in 2010, found similar results by examining vital records data for all infants born in the United States in 2002. They found that infants whose mothers smoke are 50% more likely to be born very preterm, 40% more likely to be born moderately preterm, and 20% more likely to be born in the late preterm period compared with infants whose mothers do not smoke [57].

Maternal overweight and obesity are risk factors for a host of adverse perinatal outcomes including gestational diabetes, pre-eclampsia, cesarean delivery, and stillbirth [58–61]. This study demonstrates that the risk for PTB is high in underweight women and increases further with increasing prepregnancy BMI suggesting that proper prepregnancy nutritional management may potentially influence the risk of PTB. Snowden et al. found an increased risk of PTB with increasing prepregnancy BMI, using California data from 2007 [62].

No previously published studies have included maternal geographic region as a predictor of preterm birth (PTB). The findings of this study showed that the prevalence of PTB differed by region in California (Table 2 and Fig. 4). Infants born in the San Joaquin Valley region, which consists of rural counties, comprised one-eighth of the babies born in California, and were more likely to be born preterm compared with those born in the Northern and Sierra regions. This shows the importance of including geographic information in studies of birth outcomes. Almost two-thirds of the births in the San Joaquin Valley region depended on Medi-Cal, mainly through the Medi-Cal Managed Care delivery system, and the WIC program. Therefore, preterm birth outcomes reported in this study might be a useful quality measure for policymakers to evaluate Medi-Cal health plans, and to restructure and improve prenatal care during pregnancy and to recognize women at risk for PTB.

We have recently reported the findings of a study that showed that women in the San Joaquin Valley region were 25% more likely to have low birth weight babies when compared with women in Los Angeles County [21].

Therefore, the findings of the present study can be considered to be a warning sign for policymakers to address poor birth outcomes, which are leading causes of infant morbidity and mortality. Additional studies are needed to further explore the geographic and socio-economic disparities in PTB.

Importantly, for regions where healthcare resources are limited, such as the San Joaquin Valley region, it may be more difficult to adequately manage infant morbidity arising from poor birth outcomes. This results in increased healthcare costs not only for the infant, but throughout their life. Therefore, investment in better health care for women during pregnancy may help avoid the costly complex health and social needs arising from poor birth outcomes.

Because Medi-Cal provides obstetric care for almost two-thirds of the women during pregnancy in the San Joaquin Valley region, Medi-Cal has a role in supporting early childhood development and well-being for families in financial need. Medi-Cal is uniquely positioned to identify high-risk women who have an increased need for health care and social services during pregnancy.

In 2018, Kozhimannil et al. found that delivery in rural US counties not adjacent to urban areas and loss of hospital-based obstetric services increased the risk for PTB [63]. It might be useful in the future to study these disparities across geographic regions to understand the causes and associations with PTB in more detail.

During the study period, a higher prevalence of LBW infants was found among both preterm AGA and term SGA births. Overall, SGA infants among singleton births at 23 to 41 weeks based on OE of gestational age did not change significantly, but LBW births among SGA infants declined significantly from 2007 to 2016. As Lee et al. stated in 2017, infants born SGA, whether term or preterm, carry a considerably higher risk of mortality and morbidity in the neonatal period and beyond when compared with AGA infants [3]. The risk is even greater among infants born both preterm and SGA [4].

This study showed that women with Medi-Cal as their source of prenatal care payment, used as a proxy for low income, are more likely to have a PTB than women with private insurance (Table 2). This is valuable information that can help in the development of policies to address this disparity, as almost half of the births in California are for women who are Medi-Cal beneficiaries. Women who receive first-trimester prenatal care have a small improvement in the prevalence of PTB compared with women who do not receive early prenatal care. We also found that parity is a significant predictor of PTB. Primiparous women and multiparous women with 6 to 12 prior deliveries are more likely to have a PTB (Table 2).

In 2005, Joyce et al. found that WIC is associated with an increase of 57 g in birth weight adjusted for gestation and a decline of 4.1 percentage points in the rate of SGA (*p* < 0.01) [64]. In 2016, Sonchak also observed that, women who received WIC benefits had a lower prevalence of preterm birth compared to non-WIC participants [65]. The WIC benefits apparently overpowers the effects of lower socioeconomic status of women during pregnancy with regards to preterm birth based on the obstetric estimate of gestational age. This finding affirms the value of the WIC program for pregnant women on reducing the prevalence of PTB.

There are several limitations to this study. Maternal characteristics are restricted to those contained within the California BSMF compiled by the Center for Health Statistics and Informatics of the CDPH for the period from 2007 to 2016. Maternal age, race and ethnicity, education level, smoking status during pregnancy, gestational age, and prepregnancy height and weight are self-reported. Despite these limitations and the possible effects of uncontrolled confounding variables, this analysis of almost 5 million births in the state of California provides evidence from highly diverse racial and ethnic groups and diverse socioeconomic conditions, including rural and urban regions.

## Conclusions

Evaluation of the prevalence of PTB from 2007 to 2016 in California showed a decline from 2007 to 2014 but an increase in 2015 and 2016. The prevalence of PTB remains a concern, and large disparities persist based on maternal age, race and ethnicity, maternal geographic regions, smoking during pregnancy, maternal prepregnancy BMI, education level, and parity. The significant interactions between maternal age, race and ethnicity, nutritional status and education level show various patterns and disparities in the prevalence of PTB. Women at the younger and older ends of the childbearing age range are at increased risk for PTB. Independent of maternal race and ethnicity and education level, the risk for PTB increases with maternal age. African American women living in California are more likely to have a PTB compared with white women. More research is needed to curb the increasing prevalence of PTB with maternal age. There are public health implications to the increasing prevalence of PTB in older women, because births to women of advanced maternal age increased significantly during the study period.

Maternal and child public health initiatives should focus on reducing the prevalence of PTB, currently one of the leading causes of infant and childhood morbidity and mortality, by managing the modifiable maternal characteristics, geographic disparities, and perinatal health behaviors associated with an increased risk. Our finding supports the significance of the WIC program for pregnant women on reducing the prevalence of PTB. Measures are needed to reduce the risk of PTB at advanced maternal age, and prenatal care needs to be restructured, especially among rural counties, to recognize women at risk for PTB and to treat them accordingly.

## Additional files

Additional file 1: Screening criteria to identify the study population from the California Birth Statistical Master File from 2007 to 2016. (DOCX 64 kb)

Additional file 2: Likelihood of preterm birth listed as adjusted odds ratio (with 95% confidence interval in parenthesis) for each maternal age group by maternal race and ethnic group, after accounting for confounding effects. (DOCX 21 kb)

Additional file 3: Likelihood of preterm birth listed as adjusted odds ratios (with 95% confidence interval in parenthesis) for each maternal education group by maternal race and ethnic group, after accounting for confounding effects. (DOCX 20 kb)

## Abbreviations

95% CI: 95% confidence interval
AGA: Appropriate for gestational age
AOR: Adjusted odds ratio
BSMF: Birth Statistical Master Files
CDPH: California Department of Public Health
FGR: Fetal growth restriction
LBW: Low birth weight
LGA: Large for gestational age
LMP: Last menstrual period
OE: Obstetric estimate
PTB: Preterm birth
SGA: Small for gestational age
WIC: Supplemental Nutrition Program for Women, Infants, and Children
