# Supplementary material for: Temporal trends, patterns, and predictors of preterm birth in California from 2007 to 2016, based on the obstetric estimate of gestational age
Source: Matern Health Neonatol Perinatol. 2018 Dec 12;4:25. doi: 10.1186/s40748-018-0094-0 (PMC6290518; doi:10.1186/s40748-018-0094-0)
Supplement: Supplementary file 1 — Screening criteria to identify the study population from the California Birth Statistical Master File from 2007 to 2016. (DOCX 64 kb) [file 40748_2018_94_MOESM1_ESM.docx]

**
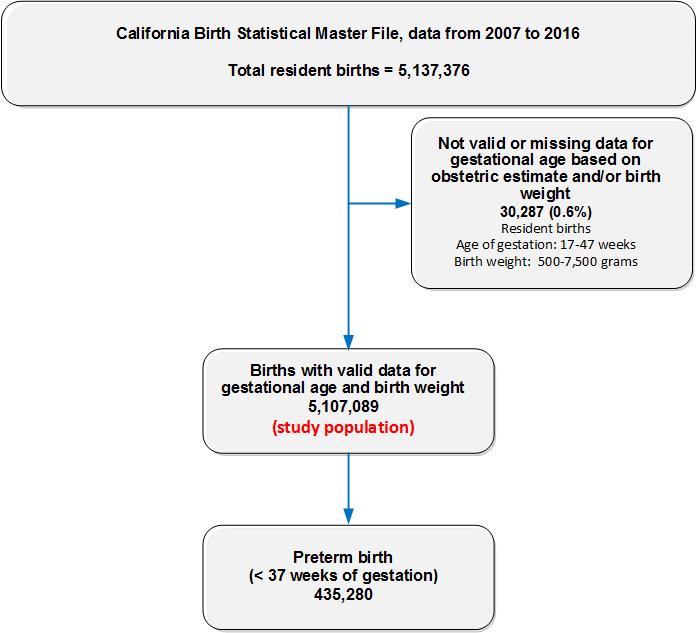
**

**Additional File 1.** Screening criteria to identify the study population from the California Birth Statistical Master File from 2007 to 2016
