# Supplementary material for: Temporal trends, patterns, and predictors of preterm birth in California from 2007 to 2016, based on the obstetric estimate of gestational age
Source: Matern Health Neonatol Perinatol. 2018 Dec 12;4:25. doi: 10.1186/s40748-018-0094-0 (PMC6290518; doi:10.1186/s40748-018-0094-0)
Supplement: Supplementary file 2 — Likelihood of preterm birth listed as adjusted odds ratio (with 95% confidence interval in parenthesis) for each maternal age group by maternal race and ethnic group, after accounting for confounding effects. (DOCX 21 kb) [file 40748_2018_94_MOESM2_ESM.docx]

**Additional File 2.** Likelihood of preterm birth listed as adjusted odds ratio (with 95% confidence interval in parenthesis) for each maternal age group by maternal race and ethnic group, after accounting for confounding effects

| **Maternal age, years** | **Hispanic** | **White** | **Asian** | **Pacific Islander** | **African America** | **Multiple Race** | **American Indian** |
| --- | --- | --- | --- | --- | --- | --- | --- |
| **<20** | **1.09 (1.07-1.11)** | 1.02 (0.97-1.07) | 1.40 (1.28-1.54) | 0.96 (0.73-1.26) | 0.97 (0.92-1.03) | 0.97 (0.87-1.08) | 0.92 (0.71-1.19) |
|  | **<.001** | 0.397 | **<.001** | 0.747 | 0.368 | 0.587 | 0.522 |
| **20-24** | Ref | Ref | Ref | Ref | Ref | Ref | Ref |
| **25-29** | **1.09 (1.07-1.11)** | **1.08 (1.05-1.11)** | 1.00 (0.95-1.05) | **1.04 (0.88-1.23)** | **1.10 (1.06-1.15)** | 1.00 (0.93-1.09) | 1.14 (0.94-1.38) |
|  | **<.001** | **<.001** | **0.939** | **0.487** | **<.001** | 0.928 | 0.077 |
| **30-34** | **1.28 (1.26-1.30)** | **1.22 (1.18-1.26)** | **1.14 (1.08-1.19)** | **1.36 (1.13-1.63)** | **1.30 (1.24-1.37)** | 1.06 (0.97-1.16) | **1.70 (1.39-2.08)** |
|  | **<.001** | **<.001** | **<.001** | **<.001** | **<.001** | 0.204 | **<.001** |
| **35-39** | **1.58 (1.55-1.61)** | **1.47 (1.42-1.52)** | **1.41 (1.34-1.48)** | **1.61 (1.30-1.99)** | **1.58 (1.49-1.67)** | **1.26 (1.14-1.39)** | **2.13 (1.67-2.72)** |
|  | **<.001** | **<.001** | **<.001** | **<.001** | **<.001** | **<.001** | **<.001** |
| **40-54** | **1.95 (1.89-2.01)** | **1.86 (1.79-1.94)** | **1.85 (1.75-1.97)** | **2.89 (2.21-3.78)** | **2.03 (1.86-2.20)** | **1.68 (1.46-1.94)** | **2.59 (1.79-3.74)** |
|  | **<.001** | **<.001** | **<.001** | **<.001** | **<.001** | **<.001** | **<.001** |

Results in bold indicate statistical significance (*p* < 0.05)

Ref = Reference group

Multivariate logistic regression models were conducted for each racial and ethnic group, controlling for maternal education, nativity, demographic region, source of prenatal care payment, first-trimester prenatal care initiation, parity, maternal smoking status, and maternal prepregnancy body mass index
