# Supplementary material for: Temporal trends, patterns, and predictors of preterm birth in California from 2007 to 2016, based on the obstetric estimate of gestational age
Source: Matern Health Neonatol Perinatol. 2018 Dec 12;4:25. doi: 10.1186/s40748-018-0094-0 (PMC6290518; doi:10.1186/s40748-018-0094-0)
Supplement: Supplementary file 3 — Likelihood of preterm birth listed as adjusted odds ratios (with 95% confidence interval in parenthesis) for each maternal education group by maternal race and ethnic group, after accounting for confounding effects. (DOCX 20 kb) [file 40748_2018_94_MOESM3_ESM.docx]

**Additional File 3.** Likelihood of preterm birth listed as adjusted odds ratio (with 95% confidence interval in parenthesis) for each maternal education group by maternal race and ethnic group, after accounting for confounding effects

| **Maternal education** | **Hispanic** | **White** | **Asian** | **Pacific Islander** | **African American** | **Multiple race** | **American Indian** |
| --- | --- | --- | --- | --- | --- | --- | --- |
| **<High school diploma** | **1.33 (1.30-1.36)** | **1.50 (1.44-1.56)** | **1.31 (1.23-1.40)** | **1.60 (1.24-2.07)** | **1.46 (1.36-1.56)** | **1.60 (1.42-1.80)** | **1.69 (1.26-2.26)** |
|  | **<.001** | **<.001** | **<.001** | **0.027** | **<.001** | **<.001** | **0.001** |
| **High school diploma** | **1.30 (1.27-1.33)** | **1.27 (1.24-1.31)** | **1.23 (1.19-1.28)** | 1.09 (0.90-1.33) | **1.30 (1.23-1.38)** | **1.32 (1.21-1.44)** | 1.28 (0.99-1.66) |
|  | **<.001** | **<.001** | **<.001** | 0.382 | **<.001** | **<.001** | 0.065 |
| **Some college/associate degree** | **1.27 (1.25-1.30)** | **1.24 (1.21-1.26)** | **1.28 (1.25-1.32)** | 1.06 (0.88-1.28) | **1.27 (1.21-1.34)** | **1.34 (1.24-1.44)** | 1.25 (0.97-1.60) |
|  | **<.001** | **<.001** | **<.001** | 0.565 | **<.001** | **<.001** | 0.084 |
| **Bachelor’s degree or higher** | Ref | Ref | Ref | Ref | Ref | Ref | Ref |

Results in bold indicate statistical significance (*p* < 0.05)

Ref = Reference group

Multivariate logistic regression models were conducted for each racial and ethnic group, controlling for maternal age, nativity, demographic region, source of prenatal care payment, first-trimester prenatal care initiation, parity, maternal smoking status, and maternal prepregnancy body mass index
